# Development and performance of PROWalk: a functional mobility person-reported outcome measure based on the PROMIS^®^ adult physical function item bank

**DOI:** 10.3389/fneur.2026.1693841

**Published:** 2026-02-23

**Authors:** Sutton B. Richmond, Laura Jacobs, Caleb Kumar, Yvonne Rumsey, Carole A. Tucker, Lars Oddsson

**Affiliations:** 1 RxFunction Inc., Eden Prairie, MN, United States; 2 Department of Global Health and Population, Harvard T.H. Chan School of Public Health, Harvard University, Boston, MA, United States; 3 Department of Physical Therapy and Rehabilitation Sciences, University of Texas Medical Branch, Galveston, TX, United States; 4 Department of Rehabilitation Medicine, University of Minnesota, Minneapolis, MN, United States

**Keywords:** functional mobility, PROMIS^®^, PROWalk, PSFS, telehealth, Walkasins^®^, wearable sensory prosthesis

## Abstract

**Introduction:**

Functional mobility, or the capacity to move safely and independently, can be evaluated using performance-based physiological tests, observational assessments, or self-report tools. This study sought to develop the PROWalk™, a PROMIS-based patient-reported outcome measure of perceived gait and balance challenges, and evaluated its performance relative to the Patient-Specific Functional Scale (PSFS) in individuals with sensory peripheral neuropathy.

**Methods:**

PROWalk was developed with expert clinician input using selected items from the PROMIS item bank. It was administered via telehealth to participants with lower-extremity peripheral neuropathy (*N* = 51; age 76 ± 4 years; 4 female) who were prescribed a wearable sensory prosthesis, alongside the PSFS, at baseline and again after 6 weeks.

**Results:**

Both measures displayed significant increases (i.e., improvements) over time with large effect sizes (PROWalk: *rrb* = −0.878; PSFS: *rrb* = −0.993), strong temporal correlations (PROWalk: *rho* = 0.684; PSFS: *rho* = 0.458), and upward linear trends for each instrument between baseline and 6 weeks. When normalized, the measures showed no bias, large limits of agreement, and moderate convergent validity (*rho* = 0.582, *p* < 0.001).

**Discussion:**

Compared with broader PROMIS Physical Function short forms, PROWalk targets mobility-specific activities relevant to gait and balance. It demonstrated significant improvements in perceived functional mobility over time and strong temporal stability, supporting its use for standardized group-level comparisons. PROWalk appears to be a valid, telehealth-compatible measure for capturing self-reported functional mobility in individuals with sensory neuropathy and may address limitations of more individualized assessment tools.

## 1 Introduction

Functional mobility refers to an individual’s physical capacity to move independently and safely in order to perform activities of daily living (e.g., transferring, housekeeping, shopping, transportation) (1). The assessment of functional mobility can be approached through various modalities, including performance-based physiological tests, observational assessments, and patient-reported outcome measures (PROM) (2). Specifically, PROM provides patient-centered insights into individual functional ability, capturing real-world participation, perceived capability, and symptom impact, which performance measures conducted in standardized settings may miss (3). The PROM construct is supported by robust regulatory guidance (4), validated measurement frameworks such as the Patient-Reported Outcomes Measurement Information System (PROMIS^®^) (5, 6), and implementation standards enabling precise, scalable, and even remote assessment (7). If PROMs are used alongside performance tests, they offer complementary information that enhances ecological validity and ensures the evaluation reflects outcomes meaningful to patients (3).

One widely used instrument is the Patient-Specific Functional Scale (PSFS), which enables individuals to identify up to five activities they find challenging due to a health condition. Each activity is self-rated based on the individual’s perceived level of difficulty. The PSFS has demonstrated strong psychometric properties, including excellent test–retest reliability (ICC = 0.848) and good construct validity, evidenced by moderate correlations with established functional outcome measures (8, 9). Finally, the PSFS is highly responsive to clinical change and offers individualized insights into patient-prioritized outcomes, making it especially useful in physical therapy and rehabilitation settings (9–11). However, the PSFS is limited by the absence of standardized metrics, the lack of discrete mobility domain identification, and its vulnerability to variability and biases. To address these limitations, we aimed to develop the PROWalk™, a standardized PROM for assessing gait and balance in adults, and apply this tool to individuals with sensory peripheral neuropathy (SPN). Ostensibly, an alternative tool that retains the personalized strengths of the PSFS while improving standardization, domain-specific resolution, and clinical applicability.

The PROMIS, which was developed with funding from the National Institutes of Health, comprises a comprehensive suite of 300 rigorously validated self-report instruments designed to assess a broad spectrum of health domains, including physical, mental, and social well-being (12, 13). Among these, the PROMIS physical function item bank has been shown to be valid and reliable to measure change in physical function in research and clinical applications.

PROMIS item banks support computer adaptive test (CAT) administration and have defined standard 4- and 8-item fixed-length, or short, forms that provide T-scores. Given that PROMIS item banks are calibrated using the item response theory (IRT), any set of items from a bank can be selected to provide “custom” short forms consisting of user-selected items that are scored on the same metric (14).

The PROMIS physical function bank consists of 165 IRT-calibrated items using a 5-point Likert scale, with most items scored on a difficulty response scale where “5” corresponded to “without any difficulty” and “1” to “unable to perform the activity.” The conceptual framework underlying PROWalk represents a reflective measurement model wherein the nine selected items serve as manifest indicators of an underlying functional mobility construct. Items were selected to span three conceptual categories within the physical function domain: (1) ambulatory capacity (e.g., walking speed, distance, stair navigation), (2) balance and postural control (e.g., standing balance, tandem walking), and (3) integrated mobility tasks (e.g., bending, overall physical activity). This selection was guided by the International Classification of Functioning, Disability and Health (ICF) framework, which recognizes mobility as encompassing changing and maintaining body position, walking and moving, and moving around using equipment (15). To date, no PROMIS Physical Function short form has been developed specifically to assess mobility-related functional impairments in individuals with gait and balance disorders. Existing standard short forms (e.g., 4a, 8a, 10a) were constructed to represent the full breadth of the physical function domain, including upper extremity function and instrumental activities of daily living, rather than targeting mobility-specific constructs. Our systematic development of the PROWalk addresses this gap by providing a targeted assessment of walking and balance-related functional activities, enabling more sensitive detection of mobility-related changes in individuals with lower-extremity impairments. This study focuses on evaluating the PROWalk as a PROMIS-based custom short-form instrument for assessing physical function and mobility while establishing its conceptual and methodological foundation. Further, we report its convergent validity with the PSFS within a population of individuals with SPN who present with gait and balance issues.

## 2 Materials and methods

### 2.1 Participants

A convenience sample of 51 participants (height: 178.8 ± 8.3 cm, mass: 96.6 ± 19.9 kg, and age: 76 ± 4 years, 4 female) provided verbal informed consent for this study. Verbal informed consent was obtained in accordance with a protocol-specific waiver of written documentation approved by Advarra IRB. This approach was deemed appropriate given the minimal-risk nature of questionnaire-based data collection and the telehealth administration format, which precluded in-person signature collection. Verbal consent was formally documented by the administering rater and included all required elements per 45 CFR 46.117(c) (2). All participants were required to provide verbal consent, be age-qualified (≥ 65 years), have a diagnosis of SPN, and use a prescribed wearable sensory prosthesis designed to replace part of the lost plantar sensory nerve function (Walkasins^®^, RxFunction Inc., Eden Prairie, MN, USA) in individuals with SPN (16, 17). The sensory deficits associated with SPN can lead to significant impairments in mobility, heightened risk of falls, and a decline in functional independence and overall quality of life (18, 19). Participants were excluded if their physical condition had changed significantly since baseline due to an injury, hospitalization, or other adverse event that disrupted their typical daily activities. Advarra IRB served as the Institutional Review Board and approved study procedures and consent in accordance with the Declaration of Helsinki.

### 2.2 Protocol

Data for this investigation were collected as part of a company-led initiative by RxFunction, Inc., under the Outcomes Program, which was developed to gain a deeper understanding of the challenges faced by individuals with SPN. The study design incorporated a baseline touchpoint (i.e., pre-intervention) followed by a post-intervention assessment conducted approximately 6 weeks later (ranging from −2 to +7 days relative to the target follow-up date).

At each assessment timepoint, participants completed a standardized battery of both quantitative and qualitative questionnaires, which evaluated key domains including demographic and anthropometric data, overall health status, and physical functionality, particularly mobility-related outcomes. To ensure procedural consistency, all follow-up assessments were administered in the same sequence as the baseline evaluations and recorded in REDCap, an electronic data capture system (20, 21).

The assessment battery was anchored by two perceived functional mobility measures: the PSFS and the PROWalk instrument (*development details below*). All examinations were conducted by a trained rater, who followed standardized administration protocols. For the PSFS, participants were asked to identify up to three activities that they were either unable to perform or found difficult to perform due to gait and balance impairments associated with peripheral neuropathy. Participants were explicitly prompted to reflect on how these impairments affected their daily functional tasks. Once activities were identified, the rater documented each activity, and participants were then asked to rate the degree of difficulty associated with performing each one using an 11-point numeric scale, where: “0” was indicative of being “Unable to perform” and “10” indicated the participant was “Able to perform at the same level as prior to the onset of the problem or injury” (12, 13). Individual activity scores were then averaged to produce a composite functional status score, ranging from “0” to “10,” with higher scores indicating better perceived function (8).

PROWalk is a custom short form comprising items selected from the PROMIS Physical Function item bank (v2.0), which has undergone rigorous development including qualitative review, cognitive interviewing, and IRT calibration (13, 22). Specifically, the development of the PROWalk followed a structured four-step process to ensure conceptual clarity, clinical relevance, and psychometric rigor: (i) *Item Compilation and Initial Screening*: Five independent reviewers evaluated the 165 items from item bank,^1^ each selecting a minimum of eight items, consistent with the typical length of PROMIS short forms, that were explicitly related to gait and postural control. Item selection followed established PROMIS methodology for developing population-targeted short forms (14, 23) and was guided by two complementary principles: (1) coverage of functional mobility activities relevant to individuals with SPN, informed by qualitative analysis of patient-reported activities during Outcomes Program interviews, and (2) inclusion of items spanning the physical function continuum to minimize floor and ceiling effects. An important part of this process was that all data analyzed here were collected after the PROWalk item selection was completed, with no subsequent modification to the selected items. All item parameters used for scoring were taken from the original PROMIS calibration and remained fixed throughout the study. Further, the selected items retain their original PROMIS IRT calibration parameters, enabling scoring on the standardized T-score metric without re-calibration. This approach is consistent with PROMIS guidelines for creating custom short forms from calibrated item banks (14).

Item selection for the PROWalk questionnaire was informed by qualitative insights gathered during patient follow-up. Specifically, we incorporated activities frequently mentioned by patients in their responses to the PSFS during Outcomes calls. These patient-reported activities reflected real-world challenges and priorities directly voiced by users of the prosthesis and were instrumental in shaping a questionnaire that is both relevant and representative of the lived experience of the clinical population. (i) *Cross-rater consolidation*: the initial selections were consolidated into a list of 24 candidate items, which were then cross-referenced across raters to identify areas of agreement and divergence. (ii) *Item reduction*: the item pool was refined to 12 items by excluding those that (a) overlapped conceptually or semantically with other items, (b) were not applicable to individuals with impaired mobility, or (c) lacked relevance to functional activities influenced by mobility limitations. (iii) *Final item selection and conceptual framework*: a final set of 9 items (see Table 1) was agreed upon by all four reviewers, based on the following conceptual criteria:

- Items should capture a broad range of physical functioning to mitigate floor and ceiling effects observed in prior user populations.
- Items must include both standing and walking activities, with greater emphasis on walking tasks.
- Selected items should reflect activities that participants have identified as meaningful during Outcomes Program interviews, specifically those they aspire to improve.

**Table 1:** PROWalk custom short form items from the PROMIS^®^ physical function item bank.

Item	PROMIS^®^ reference #	PROWalk questions
1	PFC38	Are you able to walk at a normal speed?
2	PFA30	Are you able to step up and down curbs?
3	PFA21	Are you able to go up and down stairs at a normal pace?
4	PFC39	Are you able to stand without losing your balance for several minutes?
5	PFC6r1	Are you able to walk a block (about 100 m) on flat ground?
6^†^	PFB24	Are you able to run a short distance, such as to catch a bus?
7	PFA9	Are you able to bend down and pick up clothing from the floor?
8	PFM35	Are you able to walk in a straight line putting one foot in front of the other (heel to toe) for 5 yards (5 m)?
9	GLOBAL06	To what extent are you able to carry out your everyday physical activities such as walking, climbing stairs, carrying groceries, or moving a chair?

Patient-Reported Outcomes Measurement Information System (PROMIS^®^) PROWalk questions are rated on a 5-point scale: [5] without any difficulty, [4] with a little difficulty, [3] with some difficulty, [2] with much difficulty, and [1] unable to do. ^†^Signifies a question (item) that can be removed for populations with increased mobility impairments to better align with their physical capabilities (PROWalk-8).

Item selection involved five independent reviewers during initial screening (Step i) and four reviewers for final selection (step iii). The review panel consisted of: a physical therapist specializing in neurological rehabilitation (CAT), a clinical researcher with expertise in PROMIS implementation (LO), a healthcare data analyst (SBR), and clinical coordinators experienced in patient assessment administration (LJ, YR). Disagreements were resolved through consensus discussion. This process yielded a targeted, patient-relevant instrument tailored to capture core domains of perceived functional mobility, with a focus on gait and balance. An additional single item was included on overall physical functioning (GLOBAL06) was also self-rated on a 5-point Likert scale where “5” corresponded to “completely” and “1” to “not at all” (Table 1). For all telehealth communications, raters were trained and utilized a standardized script to enhance the reproducibility of the protocol. The full PROWalk consists of nine items. An alternate 8-item version (PROWalk-8), excluding item 6 (“running to catch a bus”), is also available for populations with more severe mobility impairments where this item may not be applicable. Both versions are scored using the PROMIS scoring service. Results for the PROWalk-8 are presented in the Supplementary material.

### 2.3 Statistics

Descriptive statistics were computed to summarize the distribution of scores from the PROWalk and PSFS questionnaires. Data normality was assessed using the Shapiro–Wilk test to determine suitability for parametric testing (24). Given that PROWalk T-scores did not significantly deviate from normality, while PSFS scores violated normality assumptions, we report Wilcoxon signed-rank tests for both instruments to maintain analytical consistency. Effect sizes are reported as matched rank biserial correlations. T-scores were generated from the HealthMeasures Scoring Service for PROWalk performances (14). In the system, we selected “Custom Short Form,” “PROMIS,” “Adult,” and “Physical Function.” The item bank from which we had selected the items was PROMIS v2.0 - Physical Function, and we used the default calibration sample (PROMIS 1 Wave 1 with Extension) because the logs showed that all nine items we had chosen were used to calculate the T-scores (25, 26). Z-scores were calculated by subtracting the sample mean from each individual raw score (PSFS) or T-score (PROWalk) and dividing the result by the standard deviation, thereby standardizing values for cross-participant comparison. To evaluate changes over time and compare results between instruments, paired *t-*tests (Wilcoxon signed-rank) were conducted on both the raw data and the standardized (z-score) scores of the PROWalk and PSFS. Effect sizes were interpreted as trivial (<0.20), small (0.20–0.49), medium (0.5–0.79), or large (>0.80) (27).

Internal consistency was evaluated using Cronbach’s alpha (*α*). Interpretation of α followed the guidelines of George and Mallery (28), where values ≥ 0.90 are considered excellent, ≥ 0.80 good, ≥ 0.70 acceptable, ≥ 0.60 questionable, ≥ 0.50 poor, and < 0.50 unacceptable.

Spearman’s correlation coefficient (*rho*) was calculated to examine the relationship between the two questionnaires and evaluate the strength and direction of linear association. To assess the convergent validity between changes in mobility-specific patient-reported outcomes and perceived functional status, a correlation analysis was conducted between the z-score standardized change scores from the PROWalk and PSFS instruments. Correlation coefficients were interpreted using commonly accepted thresholds: *rho* = 0.0–0.19 (very weak), 0.20–0.39 (weak), 0.40–0.59 (moderate), 0.60–0.79 (strong), and 0.80–1.0 (very strong) (29). All analyses were performed using JASP (University of Amsterdam, Amsterdam, Netherlands, Version 0.19.3) with risk of type I error set at 𝛼 = 0.05. Graphical representations were derived using GraphPad Prism 10 (GraphPad Software, La Jolla, CA, Version 10.5.0), and results are presented with 95% confidence intervals where applicable.

## 3 Results

Descriptive data for each dependent measure are provided in Table 2. At baseline, no participants (0%) scored at the floor (minimum possible T-score) of the PROWalk, and 2 participants (3.9%) scored at the ceiling. At follow-up, floor effects remained absent (0%) while ceiling effects increased slightly to 5 participants (9.8%). Internal consistency of the PROWalk was good at baseline (Cronbach’s *α* = 0.84) and follow-up (Cronbach’s *α* = 0.86).

**Table 2:** Descriptives (mean ± standard deviation) and summary of the test of differences between baseline and follow-up.

Raw scores	Baseline	Follow-Up	Statistic	z	*p*	Effect size
PROWalk T-scores PSFS	32.85 ± 4.45 3.10 ± 1.70	36.36 ± 4.31 5.95 ± 2.17	77.500 4.000	−5.406 −6.053	<0.001 <0.001	−0.878 −0.993

Patient-Specific Functional Scale (PSFS). For the Wilcoxon test, effect sizes are given by the matched rank biserial correlation. Significant mean differences (*p* < 0.05). PROWalk 5-point Likert scale: “5” corresponded to “completely” and “1” to “not at all.” 11-point numeric scale: “0” was indicative of being “Unable to perform” and “10” indicated the participant was “Able to perform at the same level as prior to the onset of the problem or injury.” A negative matched rank biserial correlation in the configured paired differences (timepoint 1–timepoint 2) is indicative of improved physical function between timepoints.

Supplementary Table 1 presents item-level statistics including means, standard deviations, response category frequencies, and corrected item-total correlations for each PROWalk item.

**Figure 1:**
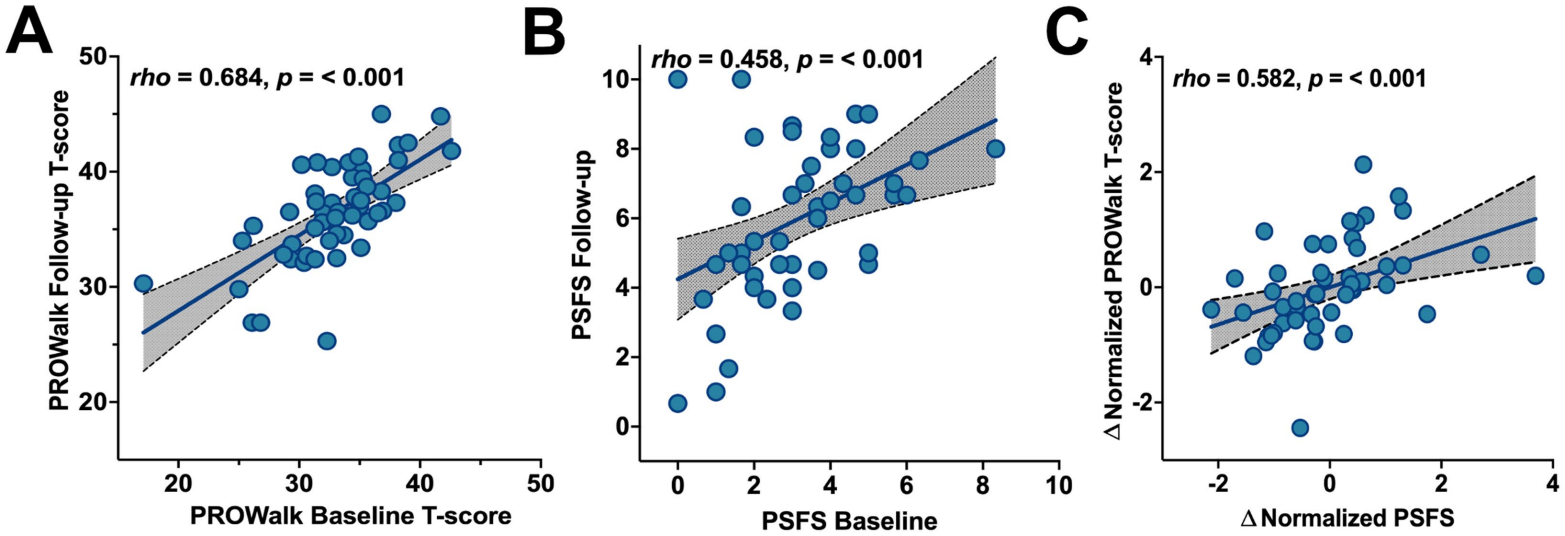
Correlations between trials and the change in scoring between instruments in persons with sensory peripheral neuropathy. Significant, positive correlations were identified for the **(A)** PROWalk (*rho* = 0.684, *p* < 0.001), **(B)** PSFS (*rho* = 0.458, *p* < 0.001), and **(C)** the change (∆) in normalized instrument scores over time (*rho* = 0.582, *p* < 0.001). All plotted values represent psychometric scores derived from Likert-scale responses and therefore do not carry physical measurement units.

Participants (*N* = 51) demonstrated significant improvements on both outcome measures over time (scattered plotted individually in Supplementary Figure 1), with large effect sizes registered (Table 2). Furthermore, both instruments showed a positive correlation over time. Figure 1 illustrates this relationship, showing an upward linear trend for each instrument (Figures 1A,B), with the PROWalk evaluation exhibiting stronger temporal consistency (*rho* = 0.684, *p* < 0.001, Figure 1A). Both instruments demonstrated responsiveness to functional change, with PROWalk detecting significant improvement (T-score increase: 32.85 to 36.36) with a large effect size (*rrb* = −0.878).

**Figure 2:**
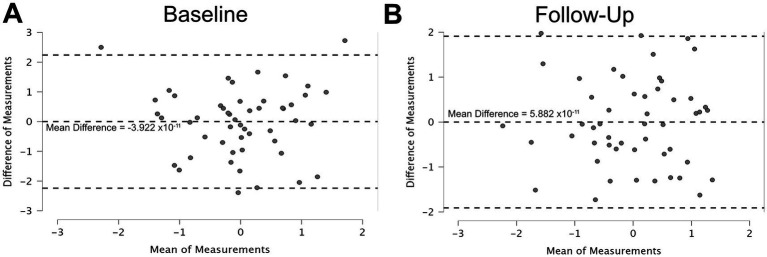
Bland–Altman plot identifying the agreement between the normalized PROWalk and PSFS instruments at **(A)** baseline and **(B)** follow-up time points. All plotted values represent psychometric scores derived from Likert-scale responses and therefore do not carry physical measurement units.

When the instrument data were normalized, the mean difference (bias) between the PROWalk and PSFS Z-scores was approximately zero (M = −3.92 × 10^−11^), indicating no systematic measurement bias between the two instruments at baseline (Figure 2A). However, the limits of agreement (LOA) ranged from −2.238 to 2.238. The follow-up agreement analysis revealed similar outcomes (Figure 2B). Furthermore, there was no significant difference (*p* = 0.725) in the normalized change between the instruments, with negligible effect sizes (0.057). A moderate positive correlation (*rho* = 0.582, *p* < 0.001) was exhibited between the change scores in PROWalk and PSFS after normalization (Figure 1C).

## 4 Discussion

The primary objective of this investigation was to evaluate PROWalk as a short-form instrument of physical function and mobility. The performance of this novel measure was examined alongside the PSFS to assess perceived functional mobility in individuals with SPN presenting with gait and balance issues. Both instruments demonstrated significant improvements in perceived functional mobility following the six-week intervention, providing evidence that PROWalk is sensitive to clinically meaningful change. Additionally, scores on the two instruments were moderately to strongly correlated from baseline to follow-up. The absence of significant score changes within each instrument over time, when normalized, further supports the validity of the PROWalk as a robust measure of perceived functional mobility. Although no systematic bias was observed between the instruments at either time point, the wide LOA suggests that individual-level discrepancies may restrict their interchangeability.

Initial findings from this investigation support the instrument’s ability to detect change without substantial floor or ceiling effects in this population. Both instruments effectively detected improvements in perceived functional mobility over time. The PSFS is well-established as a valid, reliable, and responsive outcome measure across a range of clinical populations (8, 10, 11). It has also been shown to be appropriate for between-group comparisons and for assessing change at the group level (30). Prior research has demonstrated the PSFS’s utility in tracking progressive improvement with therapeutic intervention (8), a finding corroborated in the present study. Although the PSFS exhibited slightly greater responsiveness, reflected by a larger effect size (−0.993) compared to that of the PROWalk (−0.878), this difference was minimal. The slightly greater sensitivity of the PSFS may reflect its heightened responsiveness to individual-level functional changes compared to the PROWalk. However, further research is warranted to explore this distinction more fully.

Although the PROWalk demonstrated greater temporal stability between timepoints (*rho* = 0.684, *p* < 0.001; Figure 1A) compared to the PSFS (*rho* = 0.458, *p* < 0.001; Figure 1B), the reliability of each instrument could not be formally assessed due to the interventional nature of the study design. Nevertheless, the stronger temporal consistency observed in the PROWalk suggests that it not only captures intervention-induced improvements but may also offer greater stability in tracking generalized perceived functional mobility over time. This pattern is reinforced by the PROWalk’s strong internal consistency at both timepoints, demonstrating desirable (≥ 0.80) interrelatedness among its nine items and further supporting its stability as a measure of perceived function (31). When normalized change scores were compared across instruments, a significant positive correlation emerged, indicating that participants who reported greater improvement on the PROWalk also tended to report greater improvement on the PSFS. Despite some individual-level variability, the overall pattern reflects a consistent direction of perceived functional improvement across both measures. While descriptive results indicated an upward trend in both instrument scores from baseline to follow-up, the statistical tests did not detect significant within-subject changes when scores were standardized (∆𝑍). This outcome suggests that while participants may report perceived improvement in function and quality of life, the magnitude of this change, once normalized, is statistically indistinct across the two instruments. The results also suggest the two instruments may reflect changes in related but non-identical constructs of functional status and perceived health.

The current study focused on convergent validity, assessing the relationship between PROWalk and PSFS as two measures of perceived functional mobility. The COSMIN framework recommends hypothesis testing for construct validity, where hypotheses about expected correlations with related constructs support validity (32). We hypothesized moderate-to-strong correlations between PROWalk and PSFS, given that both instruments assess patient-perceived functional mobility, albeit through different approaches (standardized vs. individualized). Future studies should examine discriminant validity by testing hypotheses about weaker correlations with measures of unrelated constructs (e.g., cognitive function, mood).

The structural validity of PROWalk was not independently assessed in this study. While the parent PROMIS Physical Function Item Bank has demonstrated sufficient unidimensionality for IRT-based scoring across multiple validation studies (22, 33), we acknowledge that confirmatory factor analysis in the SPN population would strengthen evidence for structural validity. Future studies with larger samples should examine whether gait and balance items function as distinct but correlated subdomains or as indicators of a unified functional mobility construct. While no systematic bias was observed between instruments in either interpretation of the baseline or follow-up Bland–Altman plots, the degree of individual-level disagreement underscores a limitation in their interchangeability for assessing perceived functional mobility. These findings are consistent with previous work by Abbott and colleagues on the PSFS (30), supporting the notion that such instruments may be appropriate for group-level analyses, but should be interpreted with caution when applied to individual-level clinical decision-making. The broader LOA shown in both Bland–Altman plots further emphasize the need for context-specific instrument selection. Specifically, the PSFS may offer greater sensitivity in detecting individualized perceived functional impairments, whereas the PROWalk may provide a more standardized assessment of general perceived functional status.

### 4.1 Limitations

This study has several important limitations. First, the study design did not include a control group (e.g., individuals without Walkasins use or neurotypical participants), limiting our ability to validate the findings and precluding formal reliability analysis between instruments. Additionally, the current sample was limited to individuals using a specific sensory prosthesis, which constrained both sample size and demographic diversity. While the PROWalk items are not device-specific and are intended for general assessment of functional mobility in populations with gait and balance impairments, future validation should include broader samples of individuals with lower extremity neuropathy regardless of assistive device use. Such studies would enable assessment of measurement invariance across subgroups and evaluation of the instrument’s performance in more heterogeneous populations. The interventional design, while appropriate for assessing responsiveness, precluded formal test–retest reliability assessment due to expected changes in functional status between timepoints. In addition, the presence of a potentially performance-enhancing intervention limited our ability to evaluate test–retest reliability across timepoints. As a result, a formal test–retest assessment was not feasible within this interventional design. Future studies should examine PROWalk reliability using a two-week test–retest protocol in clinically stable individuals. Moreover, the absence of a clinically validated physical performance measure, such as the Functional Gait Assessment (34, 35) or another established “gold standard” assessment of functional mobility, limited our capacity to benchmark the tested instruments. While the PSFS is recognized as a valid, reliable, and responsive outcome measure (8), its highly individualized nature may constrain its utility for direct comparison with the PROWalk. Nonetheless, the selection of the PSFS was considered the most appropriate choice given the opportunity to use it in a telehealth-based setting. Future research should focus on validating the PROWalk against more responsive and quantitative physical performance (e.g., gait) measures to better establish its reliability and clinical utility in assessing perceived functional mobility. Moreover, studies should aim to validate the PROWalk in diverse clinical populations.

Lastly, the PROWalk is designed to assess the full spectrum of mobility; however, more impaired individuals may find question six, which was originally included to minimize any ceiling effects (Table 1), unrelatable, therefore potentially limiting the aggregate score relevance across samples. Consequently, we recommend limiting the assessment to the eight applicable questions (i.e., PROWalk-8) in populations where mobility is more severely impacted. In the Supplementary Table 2 and Supplementary Figure 2, we have provided an identical analysis to the full PROWalk, displaying similar outcomes in the PROWalk-8.

### 4.2 Practical implications and future work

Following these limitations, several practical implications and future directions emerge from this work. The PROWalk offers meaningful advantages for both clinical and research use. As a PROMIS-based instrument, it yields T-scores on a standardized metric (M = 50, SD = 10), facilitating comparison across studies, settings, and populations. Its brief format, requiring only 2–3 minutes to complete, makes it feasible to administer in busy clinical environments as well as in telehealth contexts. In addition, its emphasis on mobility-specific activities may provide greater sensitivity to changes in gait and balance impairments than broader physical function measures. These attributes position PROWalk as a practical tool for monitoring patient progress and supporting individualized rehabilitation planning. Future work may focus on evaluating its responsiveness to intervention effects, validating its performance in larger and more diverse cohorts, and integrating its use within digital health platforms to expand accessibility and clinical utility.

Several directions warrant future investigation. Validation studies should (1) evaluate PROWalk performance across diverse populations with mobility impairments (e.g., post-stroke, Parkinson’s disease, geriatric), (2) assess test–retest reliability in clinically stable samples, (3) examine structural validity through confirmatory factor analysis with adequately powered samples, and (4) establish criterion validity against performance-based measures such as the Functional Gait Assessment or instrumented gait analysis. Additionally, minimal clinically important difference (MCID) values should be established to guide the interpretation of individual patient change.

## 5 Conclusion

This study makes several contributions to the PROMIS validation literature. First, it demonstrates a systematic approach to developing domain-specific custom short forms from the Physical Function item bank, guided by both psychometric principles and patient input. Second, it provides initial validity evidence for a mobility-focused instrument in a clinically relevant population with sensory neuropathy, a group underrepresented in PROMIS validation research. Third, it demonstrates the feasibility of PROMIS-based assessment in telehealth-delivered care, extending the evidence for remote PRO administration.

## Data Availability

The raw data supporting the conclusions of this article will be made available by the corresponding author upon reasonable request.

## Footnotes

1 available at: healthmeasures.net

## Supplementary material

Supplementary Figure 1 **(A)** PROWalk performance before (32.85 ± 4.45) and six weeks after (36.36 ± 4.31) wearing Walkasins^®^ in persons with sensory peripheral neuropathy. **(B)** Patient-Specific Functional Scale (PSFS) performance before (3.10 ± 1.70) and six weeks after (5.95 ± 2.17) wearing Walkasins^®^ in the same sample. The triple star indicates significance at the 0.1% level. All plotted values represent psychometric scores derived from Likert-scale responses and therefore do not carry physical measurement units.

Supplementary Figure 2 **(A)** PROWalk-8 performance before (32.91 ± 4.48) and six weeks after (36.75 ± 4.72) wearing Walkasins^®^ in persons with sensory peripheral neuropathy. The triple star indicates significance at the 0.1% level. Correlations between trials and the change in scoring between instruments across the sample. Significant, positive correlations were identified for the **(B)** PROWalk-8 (*rho* = 0.655, *p* < 0.001) and **(C)** the change (∆) in normalized instrument scores over time (*rho* = 0.561, *p* < 0.001). Agreement between the normalized PROWalk-8 and PSFS instruments was assessed using Bland–Altman plots at both baseline **(D)** and follow-up **(E)** timepoints. All plotted values represent psychometric scores derived from Likert-scale responses and therefore do not carry physical measurement units.
