# Supplementary figures and images for: Development and performance of PROWalk: a functional mobility person-reported outcome measure based on the PROMIS® adult physical function item bank
Source: Front Neurol. 2026 Feb 23;17:1693841. doi: 10.3389/fneur.2026.1693841 (PMC12967957; doi:10.3389/fneur.2026.1693841)

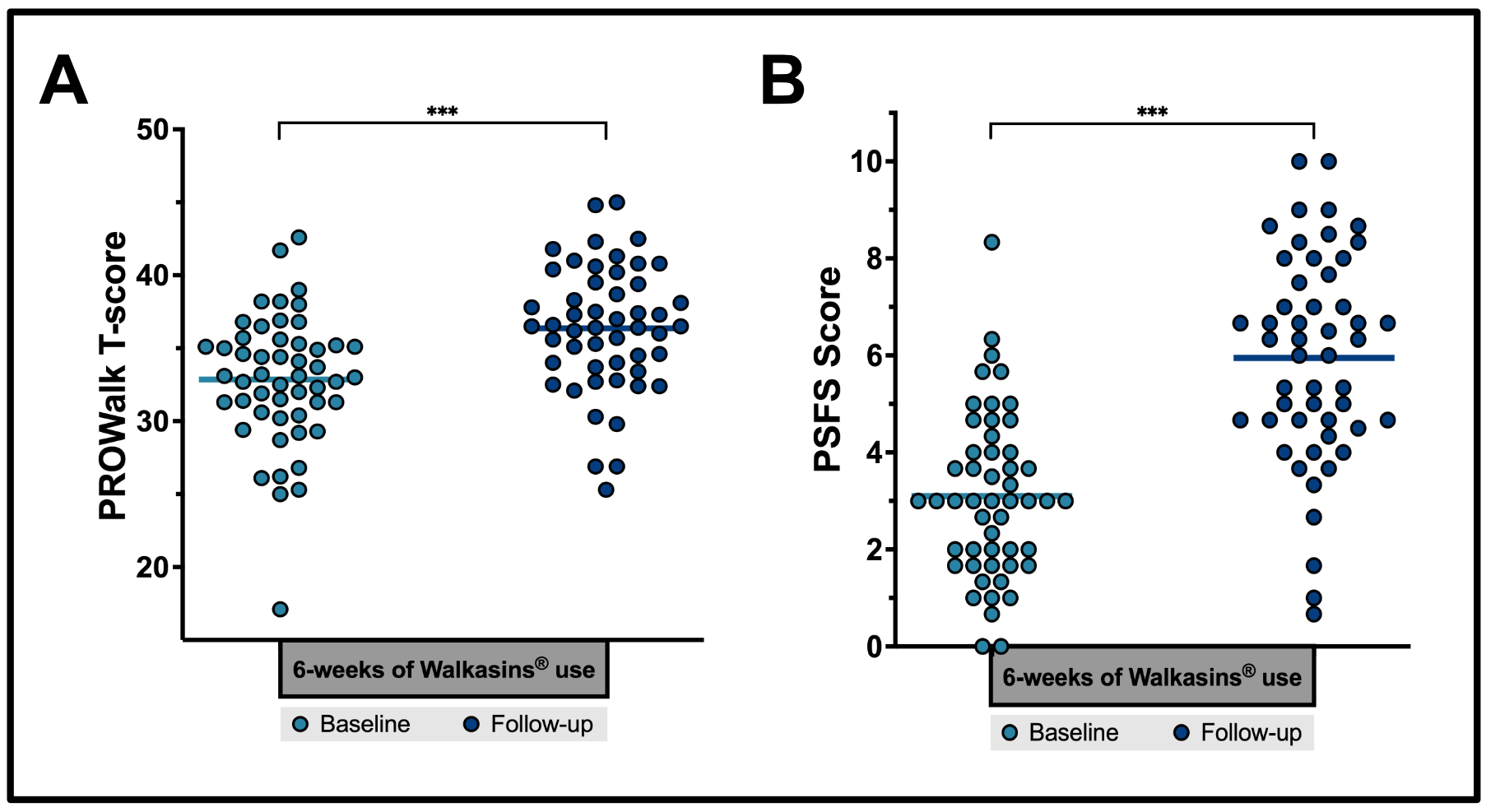

Supplement: Supplementary Figure 1 — (A) PROWalk performance before (32.85 ± 4.45) and six weeks after (36.36 ± 4.31) wearing Walkasins® in persons with sensory peripheral neuropathy. (B) Patient-Specific Functional Scale (PSFS) performance before (3.10 ± 1.70) and six weeks after (5.95 ± 2.17) wearing Walkasins® in the same sample. The triple star indicates significance at the 0.1% level. All plotted values represent psychometric scores derived from Likert-scale responses and therefore do not carry physical measurement units. [file Image_1.jpg]

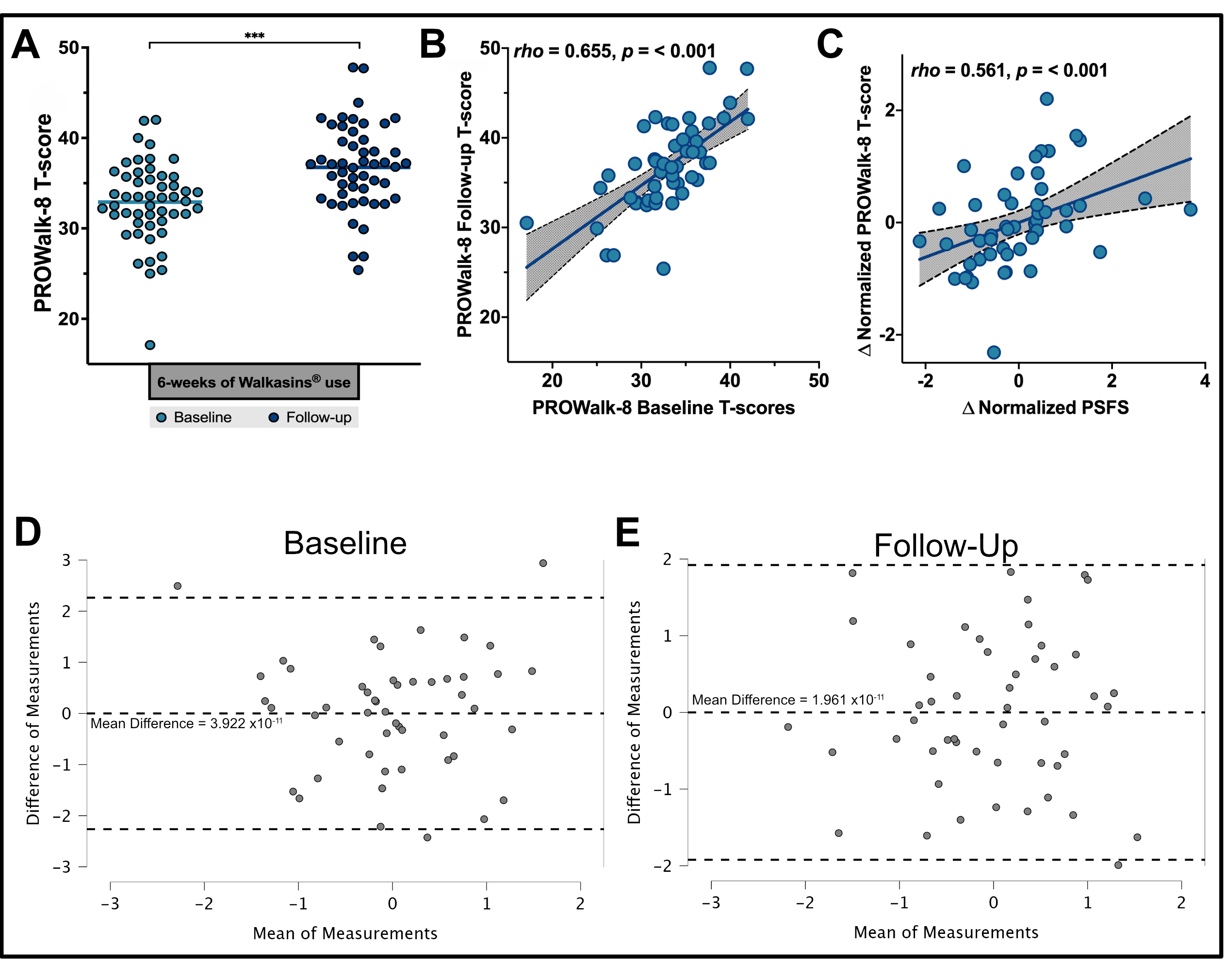

Supplement: Supplementary Figure 2 — (A) PROWalk-8 performance before (32.91 ± 4.48) and six weeks after (36.75 ± 4.72) wearing Walkasins® in persons with sensory peripheral neuropathy. The triple star indicates significance at the 0.1% level. Correlations between trials and the change in scoring between instruments across the sample. Significant, positive correlations were identified for the (B) PROWalk-8 (rho = 0.655, p < 0.001) and (C) the change (∆) in normalized instrument scores over time (rho = 0.561, p < 0.001). Agreement between the normalized PROWalk-8 and PSFS instruments was assessed using Bland–Altman plots at both baseline (D) and follow-up (E) timepoints. All plotted values represent psychometric scores derived from Likert-scale responses and therefore do not carry physical measurement units. [file Image_2.jpg]
