# Supplementary material for: Development and performance of PROWalk: a functional mobility person-reported outcome measure based on the PROMIS® adult physical function item bank
Source: Front Neurol. 2026 Feb 23;17:1693841. doi: 10.3389/fneur.2026.1693841 (PMC12967957; doi:10.3389/fneur.2026.1693841)
Supplement: Supplementary file 3 [file Table_1.pdf]

Supplementary Material

Supplemental Table 1. Item-level descriptives (mean ± standard deviation), response category frequencies, and item-total correlations for the PROWalk assessment.

Baseline

Item	*Score Frequency (5)*	*(4)*	*(3)*	*(2)*	*(1)*	*Mean ± Standard Deviation*	*Item-Total Correlations*
1	0	7	23	15	6	2.61 ± 0.87	0.637***
2	3	10	25	8	4	3.00 ± 0.97	0.601***
3	0	7	15	19	10	2.37 ± 0.96	0.652***
4	4	9	16	13	9	2.73 ± 1.18	0.709***
5	8	9	16	12	6	3.02 ± 1.24	0.775***
6†	0	1	6	8	36	1.45 ± 0.78	0.511***
7	5	10	21	14	1	3.08 ± 0.98	0.617***
8	0	2	15	11	23	1.92 ± 0.96	0.496***
9	3	15	12	19	2	2.96 ± 1.04	0.698***

Follow-up

Item	*Score Frequency (5)*	*(4)*	*(3)*	*(2)*	*(1)*	*Mean ± Standard Deviation*	*Item-Total Correlations*
1	9	17	20	0	5	3.49 ± 1.10	0.598***
2	7	22	16	6	0	3.59 ± 0.88	0.655***
3	2	10	23	9	7	2.82 ± 1.03	0.841***
4	8	19	12	5	7	3.31 ± 1.26	0.525***
5	19	17	9	3	3	3.90 ± 1.15	0.712***
6†	0	1	4	15	31	1.51 ± 0.73	0.398***
7	12	19	15	5	0	3.75 ± 0.93	0.649***
8	1	14	11	12	13	2.57 ± 1.20	0.559***
9	5	14	18	14	0	3.20 ± 0.96	0.752***

*Note. *** Significant item-total correlations ($p < .001$). Sample correlation coefficients were based on Spearman's rho calculations.*

Note. PROWalk 5-point Likert scale: "5" corresponded to "completely" and "1" to "not at all.

Note. † Signifies a question that can be removed for populations with increased mobility impairments to better align with their physical capabilities (PROWalk-8).
