# Supplementary material for: Development and performance of PROWalk: a functional mobility person-reported outcome measure based on the PROMIS® adult physical function item bank
Source: Front Neurol. 2026 Feb 23;17:1693841. doi: 10.3389/fneur.2026.1693841 (PMC12967957; doi:10.3389/fneur.2026.1693841)
Supplement: Supplementary file 4 [file Table_2.pdf]

Supplementary Material

Supplemental Table 2. Descriptives (mean ± standard deviation) and summary of the test of differences between baseline and follow-up of the PROWalk-8.

Measure	Baseline	Follow-Up	Statistic	z	*p*	*Effect Size*
PROWalk-8 T-scores	32.91 ± 4.48	36.75 ± 4.72	71.500	-5.464	<0.001	-0.888

Note. For the Wilcoxon test, effect sizes are given by the matched rank biserial correlation. Significant mean differences (p < .05).

Note: A negative matched rank biserial correlation in the configured paired differences (timepoint 1 – timepoint 2) is indicative of improved physical function between timepoints.
